# Severe Heart Dysfunction Caused by Leptospiral Myocarditis

**DOI:** 10.4269/ajtmh.18-0377

**Published:** 2018-11

**Authors:** Marco Zuin, Gianluca Rigatelli, Loris Roncon

**Affiliations:** 1 Section of Internal and Cardiopulmonary Medicine, Department of Medical Science, University of Ferrara, Ferrara, Italy;; 2 Department of Cardiology, Rovigo General Hospital, Rovigo, Italy;; 3 Department of Interventional Cardiology, Rovigo General Hospital, Rovigo, Italy

A 32-year-old man with no previous history of cardiovascular disease spent 12 days in the Kerala region of India as a tourist. During this trip, he had two hand and face contacts with stagnant water while kayaking in the backwaters near Alleppey city. Three days after he came back to Italy, he developed fever (37.5°C), dyspnea, weakness, nausea, and chest pain. On the fourth day he was hospitalized. On admission, the C-reactive protein was elevated (4.5 mg/dL), and the patient had a white blood cell count of 12 × 109/L, high N-terminal pro-brain natriuretic peptide (3,534 pg/mL), and slightly elevated troponin I (80 ng/mL) and procalcitonin (0.6 ng/mL). His electrocardiogram showed sinus tachycardia and nonspecific left ventricular repolarization abnormalities (Figure 1A). Chest X-ray revealed new cardiomegaly (Figure 2A) compared with a previous examination 1 year before (Figure 2B). Computed chest tomography confirmed the cardiomegaly; transthoracic echocardiography showed impaired systolic function (ejection fraction, 25%), biventricular dilation, and global hypokinesis; no vegetations or mural thrombi were detected. Serological tests for hepatitis B and C viruses, human immunodeficiency virus-1, Coxsackie B virus, parvovirus B19, adenoviruses, herpes simplex viruses, cytomegalovirus, varicella virus, rabies virus, West Nile virus, and legionella were negative; a malaria test was negative. Because leptospirosis is endemic in Kerala, leptospirosis was tested for by using an immunoglobulin M enzyme–linked immunosorbent assay (ELISA) (Serion-virion, GmbH, Würzburg, Germany) and a microscopic agglutination test. The former test demonstrated the presence of anti-leptospira IgM by ELISA (1:2,560), whereas the latter showed a significant agglutination (1:640), respectively. The diagnosis was further confirmed by polymerase chase reaction which detected the presence of *Leptospira interrogans* DNA in urine. Cardiac magnetic resonance imaging demonstrated hyper-intensive areas on T2 sequences with fat saturation and myocardial late gadolinium enhancement in the mid-basal anteroseptal and lateral segments (Supplemental Videos 1 and 2). The patient received ceftriaxone (2 g daily) for 14 days intravenously and supportive care. He was discharged after 20 days with normalized blood tests and ECG (Figure 1B). Despite being rare in western countries, myocarditis is a known early and severe manifestation of leptospirosis.^1^ Myocarditis should be considered during serial history and physical examinations and investigated appropriately (electrocardiography and chest X-ray) when leptospirosis is suspected.^2^

**Figure 1.**
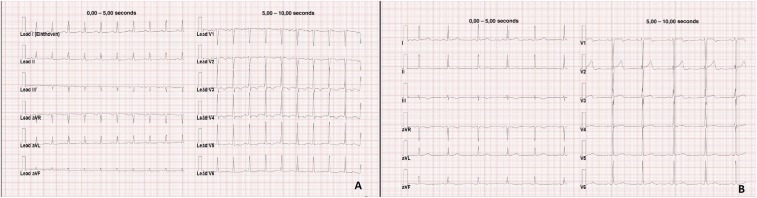
(**A**) ECG at admission showing sinus tachycardia and nonspecific left ventricular repolarization abnormalities; (**B**) ECG at discharge demonstrating sinus rhythm and nonspecific repolarization abnormalities. This figure appears in color at www.ajtmh.org.

**Figure 2.**
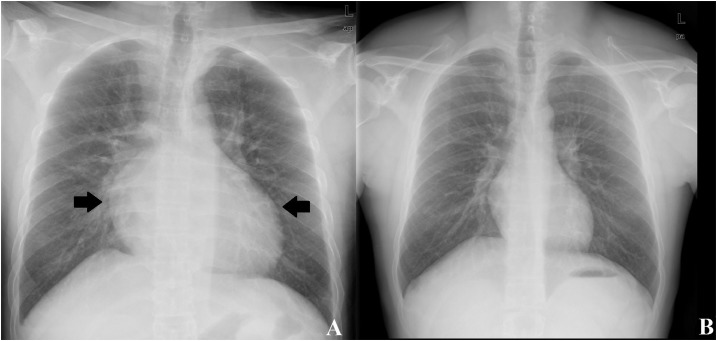
(**A**) Chest X-ray performed at admission showing severe cardiomegaly; (**B**) chest X-ray performed 1 year before the admission after a chest trauma which has been used for comparison.

## Supplementary Material

Supplemental videos
