# Supplementary material for: Severe Heart Dysfunction Caused by Leptospiral Myocarditis
Source: Am J Trop Med Hyg. 2018 Nov;99(5):1108–9. doi: 10.4269/ajtmh.18-0377 (PMC6221234; doi:10.4269/ajtmh.18-0377)
Supplement: Supplementary file 3 [file tpmd180377.SD3.pdf]

The following are supplemental materials and will be published online only

Supplemental Video 1: Cardiac MRI T2 weighted sequences with fat saturation demonstrating hypersignal areas in the mid-lateral segments

Supplemental Video 2: Delayed myocardial enhancement (MDE) with an inversion time delay of 260 ms showing myocardial late gadolinium enhancement in the mid inferior and septal segments of the left ventricle.

Note: Supplemental Videos 1 and 2 will be available online in final publication
